# Acute Toxicity and Antioxidant and Antibacterial Activities of *Kyllinga polyphylla* Willd. ex Kunth, Cyperaceae Family

**DOI:** 10.1155/2024/3543828

**Published:** 2024-01-18

**Authors:** Van-Ay Nguyen, Thi-Hang Phung, Thi-Diem-Trang Kieu, Trong-Hong-Phuc Nguyen

**Affiliations:** ^1^Can Tho University, Can Tho City, Vietnam; ^2^Can Tho Medical College, Can Tho City, Vietnam

## Abstract

*Kyllinga polyphylla* Willd. ex Kunth. (KP) is a wild herb commonly distributed in the Mekong Delta, Vietnam. This study was carried out to evaluate the antibacterial and antioxidant activities and acute toxicity of KP before conducting studies at the in vivo level. All parts of KP had the free radical scavenging capacity of DPPH, in which the root methanol extract had the best antioxidant capacity (EC_50_ = 9.54 ± 0.37 *μ*g/mL). Most of the extracts had a wide range of antibacterial spectra. The methanol and ethanol extracts (200 mg/mL) have ability to resist eight common bacterial strains (including *Staphylococcus aureus*, *Listeria innocua*, *Bacillus subtilis*, *Bacillus cereus*, *Escherichia coli*, *Salmonella* sp., *Pseudomonas aeruginosa*, and *Enterococcus faecalis*), which is equivalent to the antibacterial activity of amoxicillin and tetracycline at a concentration of 1 mg/mL. KP extracts did not cause death at a dose of 5000 mg/kg body weight and did not significantly change the biochemical, hematological, as well as histological structures of internal organs in toxicity-tested mice in comparison with the control. The research results showed that KP should be more interested in research that supports disease treatment, synthetic extraction of antibiotics, or other in vivo studies.

## 1. Introduction

Oxidative stress is a state of imbalance in the body when free radicals (ROS) exceed the body's ability to regulate [1]. The ROS produced in excess can be the cause of many dangerous diseases such as cancer, asthma, hepatitis, immunodeficiency, aging, neurodegeneration, ischemia, atherosclerosis, arrhythmia, hypertension, and diabetes [2, 3]. In addition, the mass use and abuse of synthetic antibiotics in the treatment of diseases have led to a significant increase in the phenomenon of drug tolerance or resistance [4]. This is the main cause of increased failure of treatment with high mortality and treatment costs [5, 6]. Therefore, the research for new plant resources with antibacterial and antioxidant properties is necessary for the development of a safer drug or functional food for human consumption [7, 8].

Most plants with high antibacterial and antioxidant properties have been shown to contain many bioactive compounds [9]. The medicinal value of these plants is reflected in the bioactive phytochemicals that create certain physiological effects on the human body. When compared to often employed synthetic chemotherapeutic drugs, the most significant characteristic of natural bioactive components is that they are more effective with minimal or no side effects [10]. Therefore, preliminary screening of phytochemical composition is necessary to detect and develop new therapeutic agents with improved efficacy [11].

Currently, many studies on the biological activity of plants have been carried out, in which plants belonging to the family Cyperaceae have been recorded with about 5,000 species (in 104–122 genera) and have been described [12]. According to research by Martins et al. [13], many species of the Cyperaceae family have been scientifically demonstrated for biological activities in both in vivo and in vitro studies. However, many species of the genus *Kyllinga* (there are about 40 species distributed worldwide [14]) have remained largely untested for their medicinal potential in a scientific manner and therefore require additional research. Therefore, this study was conducted to investigate the phytochemical composition and evaluate the antibacterial and antioxidant activities of the KP extracts, with the aim of the following in vitro and in vivo studies to add new bioactive plant information to the current medicine.

## 2. Materials and Methods

### 2.1. Experimental Materials

Plant samples were collected according to the Vietnam Pharmacopoeia Vol. 5 [15]. Plant samples were identified by plant taxonomists (Dr. Phung Thi Hang and Assoc. Prof. Dang Minh Quan) based on morphological characteristics and anatomical structure, as described by Nguyen[16]. Samples after identification were stored at the Animal Physiology Lab, Department of Biology, School of Education, Can Tho University. The parts studied for making the extracts included (1) roots, (2) rhizomes, (3) aerial parts (leaves and flowers), and (4) the whole plant.

### 2.2. Preparation of Plant Extracts

#### 2.2.1. Hot Water Extract

The dried sample (using a sample drying cabinet set at 50°C) was ground into powder and sieved through a sieve with a pore diameter of 0.1 mm. The powder was weighed and added to distilled water at a rate of 1 : 20 g/ml (10 g of powder). The mixture was boiled (90°C) for 20 min, and then, the extract was cooled and filtered through a filter paper (15–20 *μ*m pore size filter paper). A rotary evaporator (SCI100, Scilogex Pro, USA) was used to evaporate the mixture at 50°C to obtain the extract. The obtained extracts were stored at 4°C. The experiment was performed 3 times [17].

#### 2.2.2. Ethanol and Methanol Extract

The medicinal powder was soaked in methanol (10% w/v, powder: methanol) and ethanol (10% w/v, powder: ethanol) for 3 h and then filtered through filter papers (15–20 *μ*m pore size). After filtering, the solution was then soaked with methanol or ethanol in ratio 1 : 10 for 3 h (repeated 3 times). The extracts were combined, and then, the mixture was evaporated using a rotary evaporator (SCI100, Scilogex Pro, USA) at 50°C [17]. The final product was stored at 4°C.

#### 2.2.3. Extraction Efficiency

Extraction efficiency was determined as Extraction efficiency=(Extract weight/Powder weight) × 100.

### 2.3. Preliminary Screening of Phytochemicals

Phytochemical compounds from root, rhizome, aerial parts, and whole plant of KP were determined according to the standard methods for the qualitative determination of organic compounds in plants [18–21]. Ten (10) g of dry powder was weighed and placed into a 250 mL conical flask and then soaked with 200 mL of solvent (water, methanol, ethanol, diethyl ether, and chloroform) for 12 h on an orbital shaker at room temperature. The extracts were filtered with a filter paper (20 *μ*m filtered pore) and used for the determination of alkaloids, carbohydrates, flavonoids, phenols, proteins and amino acids, saponins, sterols, tannins, terpenoids, gums, glycosides, phlobatannins, xanthoproteic, anthocyanins, coumarins, essential oil, carotenoids, diterpenes, resins, betalains, and cardiac glycosides.

### 2.4. Antioxidant Assay

The antioxidant capacity of the extract was quantified by the free radical scavenging activity of DPPH. Water, methanol, ethanol extracts of root, rhizome, aerial parts, and whole plant of KP and vitamin C (control) were dissolved in methanol to a range of concentrations (0–200 *μ*g/mL). DPPH was diluted in methanol at a concentration of 500 *μ*g/mL. The amount of 950 *μ*L of the extract was mixed with 50 *μ*L of DPPH solution (500 *μ*g/mL). The mixture wells were shacked and incubated in the dark for 30 minutes at room temperature. The sample was then measured for absorbance at 517 nm. The experiment was repeated 3 times [17].

(1) $$\begin{array}{c} \text{Free radical neutralizing performance }\left(\%\right)=\left(\frac{\left(A_{\text{Control}}-A_{\text{Extract}}\right)}{\left.A_{\text{Control}}\right)}\right)\times 100. \end{array}$$

The EC50 index, which measures a substance's ability to reduce 50% of the free radicals in DPPH present in a solution, reflects the extracts' antioxidant capability.

### 2.5. Antibacterial Assay

The antibacterial activity of the extract was determined based on the formation of a sterile ring around the agar well [17]. We applied 50 *μ*L of a 10^6^ CFU/mL bacterial solution equally on agar and let it drain for 15 minutes. We took 20 *μ*L of solutions, respectively: positive control (amoxicillin, tetracycline, erythromycin, lincomycin, and streptomycin at concentration of 1 mg/mL), negative control (dimethyl sulfoxide 30%), and extract with 5 different concentrations (10, 50, 100, 150, and 200 mg/mL) into each well on a Petri dish. The plates were left to stand for 15 min and incubated at 37°C for 24 h before measuring the diameter of the sterile ring. The experiment was repeated 3 times.

The antibacterial ability of KP was evaluated through its ability to resist strains of Gram-positive bacteria (*Staphylococcus aureus* ATCC25923™, *Listeria innocua*, *Bacillus subtilis* ATCC23857™, *Enterococcus faecalis*, and *Bacillus cereus* ATCC14579™) and Gram-negative bacteria (*Escherichia coli* ATCC25922™, *Salmonella* sp., and *Pseudomonas aeruginosa*).

### 2.6. Acute Toxicity Experiment

#### 2.6.1. Animal Test

Healthy laboratory Swiss mice (*Mus musculus* var albino) of both sexes (28–32 g) were purchased from the Stem Cell Institute (Ho Chi Minh City, Vietnam) for toxicity testing. The mice were acclimatized to laboratory captivity (12-hour light/12-hour dark cycles) for one week, free-eating. Mice were fasted for 24 h before toxicity testing.

#### 2.6.2. Acute Toxicity Test

Acute toxicity tests were performed according to the method of Anon [22]. Experimental mice were divided into 4 treatments, each treatment consisted of 12 mice (6 males: 6 females). Before the test, mice were fasted for 24 h, but were allowed to drink water freely. Mice received a single oral extract with doses for each group including treatment 1 (distilled water) and treatments 2, 3, and 4 with doses of 1000 mg/kg, 2000 mg/kg, and 5000 mg/kg, respectively.

After taking the extract, the experimental mice were monitored for the following expression and recorded the status and survival rates at 3 h, 6 h, 12 h, 24 h, 48 h, and 72 h. After 72 hours of follow-up, the mice were euthanized, and blood and internal organs were taken to assess the toxicity of the extract.

#### 2.6.3. Indicators for Monitoring Symptoms of Toxicity

Symptoms of poisoning observed include changes in body skin color, state of the nervous system (sleep, coma, convulsions, death, muscle tremors, grooming, and balance), respiratory rate, health of the digestive system (diarrhea, digestive activity, food intake, and salivation), and excretion (urine output) [23–26].

#### 2.6.4. Hematological and Biochemical Parameters

After weighing, mice would be humanely killed under general anesthesia. Blood collected from the heart was tested for hematological parameters (using a CELL-DYN Ruby Automated Hematology Analyzer, Abbott, USA) and biochemical indicators including AST (aspartate transaminase), ALT (Alanine transaminase), and uric acid (using the Cobas Clinical Chemistry Automatic Analyzer, La Roche Ltd., Japan).

#### 2.6.5. The Organ-Body Index

Mice were then dissected for internal organs including the heart, liver, lungs, kidney, spleen, testis, or ovary. The internal organs were washed with physiological saline and dried with a blotting paper and then weighed in absolute terms. The organ-body indexes were calculated using the formula:

Organ-body index (%) = organ weight (g)/animal weight (g) [27].

#### 2.6.6. Histological Assessment of Disease

Tissue samples of organs were fixed in Bouin's solution for histological evaluation. Tissue samples were cut using a microtome (Accu-Cut® SRM™ 200, Sakura, Japan) to 5 *μ*m thin slices and stained with hematoxylin-eosin (HE). Histological samples were observed with an Olympus CX23 microscope, and histological images were taken with a digital camera with ToupView software [23].

#### 2.6.7. Statistical Analysis

The data were stored and analyzed by using SPSS 22.0 software (SPSS Inc, USA). The difference between the treatment groups and the obtained values were compared by one-way ANOVA statistic with Duncan's post-hoc test at 95% confidence. The data are presented as the mean ± SD.

## 3. Results and Discussion

### 3.1. Preliminary Phytochemical Screening

In KP, there are 19 existed compounds including alkaloids, carbohydrates, cardiac glycosides, flavonoids, phenols, proteins/amino acids, saponins, sterols, tannins, terpenoids, gums, glycosides, xanthoproteic, anthocyanins, coumarins, essential oils, diterpenes, resins, and betalains (Table 1). Among the examined species, the rhizomes had 16 compounds, the root parts had 15 compounds, and the aerial parts had 14 compounds. There were 10 compounds present in all 3 parts of the plant including carbohydrates, cardiac glycosides, flavonoids, phenols, tannins, xanthoproteic, coumarin, essential oil, diterpenes, and betalains. However, there are some compounds that were only present in one part, such as alkaloids only found in the roots and saponins in the stem. Compounds extracted in different solvents also gave different results, specifically as follows: methanol (14 compounds) > ethanol (13 compounds) > water (12 compounds) > chloroform (11 compounds) > diethyl ether (7 compounds). This result is similar to studies on the chemical composition of species in the same genus as *K. nemoralis*, *K. triceps*, and *K. monoceps*. *K. nemoralis* had glycosides, flavonoids, tannins, carbohydrates, triterpenoids, proteins, amino acids, and phenolics [28, 29]; *K. triceps* had steroids, alkaloids, glycosides, phenolics, flavonoids, saponins, tannins, and amino acids [9, 30]; *K. monoceps* contained coumarins, saponins, steroids, tannins, and terpenoids [9].

In medicine, the medicinal value of plants is highly dependent on the chemical compositions of that medicinal plant [31, 32]. Numerous studies have demonstrated that coumarin has the ability to scavenge free radicals and metal ions [33]. Flavonoids have anti-inflammatory and antioxidant properties [34] and anticancer activity [35]. Phenols have been shown to have anticancer [36], anti-inflammatory [37], antibacterial [38], and antioxidant activities [39]. Tannins have anti-inflammatory, antidiarrheal [33], and anti-irritant properties [40]. Alkaloids have pharmacological activities such as antibacterial [41], antiarrhythmic, and relief in pain [42] and lower blood glucose [43]. Saponins show many biological activities such as anti-inflammatory, hypocholesterolemic and immunostimulating [44], antibacterial [45], and antidiabetic [46]. The presence of biologically active substances such as phenol, coumarin, alkaloids, steroids, flavonoids, tannins, and carbohydrates prevents and protects the body from many diseases [42, 47] which shows pharmacological value material of KP.

### 3.2. Extraction Efficiency

Several parts of the plant, after washing, were dried to determine the moisture content of the medicinal herbs. The results showed that the moisture content of these parts increased gradually from rhizome (73.40 ± 0.80%) to whole plant (74.16 ± 1.39%), root (75.58 ± 1.23%), and rhizome (81.40 ± 0.33%) (*p* < 0.05).

Researchers have increasingly tended to investigate new sources of natural medicine. Many studies related to the evaluation of solvent-based extracts obtained from plants, and their biological activities have received more attention in recent years [48]. Therefore, in this study, the extract of KP species was evaporated with 3 solvents of methanol, ethanol, and water.

Statistical results in Table 2 show that roots had the lowest extraction efficiency, about 1-2%, while the remaining parts included rhizomes, aerial part, and the whole plant which had a higher extraction efficiency. Ethanol gave the lowest extraction efficiency among the extraction solvents, with only a 1–4% yield. Aqueous and methanol solvents had higher extraction yields, from 10 to 15%. The amount of extract was different in distinct parts of the plant, depending on the diverse extraction solvents. Therefore, it is necessary to detect suitable solvents for each type of medicinal plant as well as its different parts to receive the maximum amount of extract.

### 3.3. Antioxidant Activity

The antioxidant activity of biologically active compounds is demonstrated by maintaining the cell structure and function by scavenging free radicals, inhibiting lipid peroxidation reactions, and preventing other oxidative stress [49]. Many studies have proven that antioxidants can prevent chronic diseases such as cancer, diabetes, and cardiovascular disease [50]. Thus, the antioxidant capacity of the extracts obtained from KP was evaluated by the free radical scavenging efficiency of DPPH through the EC_50_ value (*μ*g/mL).  Table 3 indicates that all extracts from parts of KP have antioxidant activity. The DPPH free radical scavenging efficiency of the extracts is arranged in the following order: vitamin C, RMe, REt, RhEt, WEt, APEt, WMe, RhMe, WHw, RhHw, APMe, RHw, and APHw.

Among all the investigated extracts, the methanol root extract had the best DPPH free radical scavenging ability (EC_50_ = 9.54 ± 0.37 *μ*g/mL) with a concentration only 4.77 times higher than vitamin C. In each part, most ethanol extracts had superior antioxidant capacity compared to water and methanol extracts. The DPPH free radical scavenging results of KP in this study are similar to those of some species in the same genus such as *K. nemoralis* [29, 51] and *K. monocephala* [52]. According to the above analysis, all parts of KP species have respectable antioxidant capacity, in which the ethanol extract from aerial parts had great potential for application with high antioxidant capacity and extraction efficiency.

### 3.4. Antibacterial Activity

**Figure 1:**
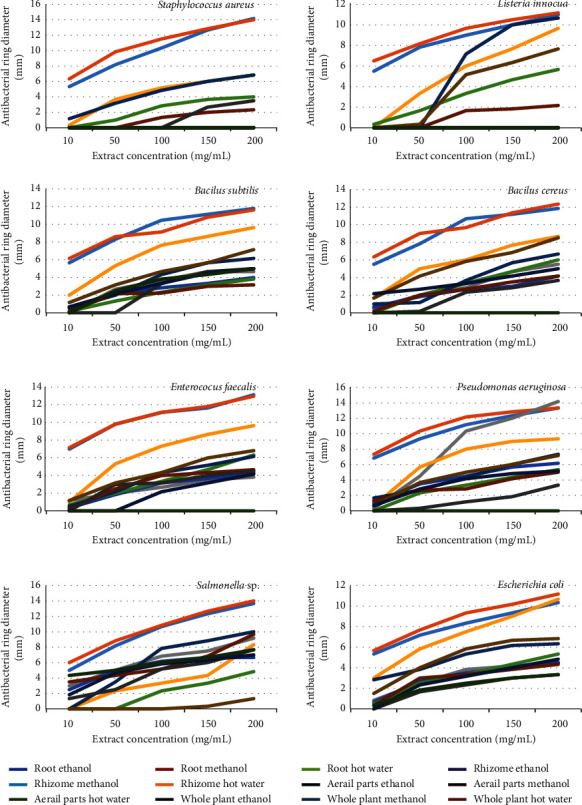
Antibacterial diameters of KP extracts at different concentrations.

The results of the study on the antibacterial ability of KP extracts through the antibacterial diameter showed that they were dependent on the type of extract and the concentration of the extract. The antibacterial ability of the extract was higher when present at high concentrations in agar Petri dish (Figure 1). The secondary metabolites found in KP extracts (Table 1) have been illustrated that they have biologically active roles such as antioxidant, antibacterial, antilarval, antiviral, antifungal, and insecticidal activities along with other beneficial activities found to be associated with species survival [53].

Antimicrobial compounds could be classified according to their spectrum of activity, bacteriostatic action, and mechanism of inhibition. Based on the spectrum of activity, antibacterial compounds were divided into broad spectrum (capable of inhibiting the growth of Gram-negative and Gram-positive bacteria) and narrow spectrum (can only inhibit the growth of Gram-negative or Gram-positive bacteria) [54]. Results in Table 4 show that KP had broad-spectrum antibacterial compounds because these extracts were resistant to both Gram-positive bacteria (*S. aureus*, *L. innocua*, *B. subtilis*, and *B. cereus*) and Gram-negative bacteria (*E. coli*, *Salmonella* sp., *P. aeruginosa*, and *E. faecalis*).

In consideration of the antibacterial ability of the extracts at a concentration of 200 mg/mL, it was found that most of the extracts were able to inhibit bacteria. The antibacterial activity of the extract was determined according to Bonomo et al. [55] through antibacterial diameter: 0–9 mm: low; 10–14 mm: moderate; and >15: high. The results showed that KP extract was only moderately antibacterial. Some plant extracts did not show antibacterial activity (Figure 1, Suppl. 1): whole plant hot water extract (did not show resistance to *S. aureus*, *L. innocua*, *B. cereus*, *P. aeruginosa*, and *E. faecalis*), whole plant ethanol extract (not resistant to *S. aureus*), aerial part hot water extract (not resistant to *L. innocua*), and whole plant methanol extract, aerial part methanol extract, and hot water root extract (not resistant to *S. aureus*, *L. innocua*).

Meanwhile, ethanol and methanol root extracts showed more prominent antibacterial activity than other extracts with greater diameter of inhibitory zones than amoxicillin (1 mg/mL) and equivalent to tetracycline (1 mg/mL). Subsequently, the aerial part ethanol extract recorded higher antibacterial activity than amoxicillin (1 mg/mL) and other extracts. Root extracts using methanol and ethanol as solvent had very good oxidation resistance (Table 3), and it had demonstrated excellent antimicrobial properties; however, it had the lowest extraction efficiency (Table 2). The ethanol extract from the aerial part of KP with a high antibacterial effect, high antioxidant capacity, and higher yield of extract than the roots will be a potential source of raw materials for medicine exploitation and development.

### 3.5. Acute Toxicity

Plant species with bioactive compounds are commonly used for medicinal purposes. However, some are very active and these compounds cannot be used therapeutically because of their toxic, carcinogenic, and mutagenic properties [56]. These previous findings emphasized the need for a thorough safety assessment of herbal preparations [57]. From the research results on antibacterial and antioxidant capacity of extracts from parts of KP, it was assumed that the aerial part ethanol extract of this species had high potential and can be applied in medicine. This investigation, hence, performed the acute toxicity study of KP aerial part ethanol extract as a premise for the next in vivo studies.

#### 3.5.1. Expression of Toxicity and Determination of LD_50_ Value

Giving mice a single oral extract with doses of 1000 mg/kg, 2500 mg/kg, and 5000 mg/kg, respectively, did not cause death in 72 hours of the experiment period. Therefore, the LD_50_ value was not determined (LD_50_ > 5000 mg/kg). According to the Global Chemical Classification and Labeling System (GHS), substances with an oral LD_50_ value >5000 mg/kg are considered relatively safe after acute toxicity [58]. Clinical signs of toxicity including change in skin, coma, convulsion, diarrhea, digestion, drowsiness, eye color, food intake, general physique, grooming, limp, rate of respiration, salivation, sedation, seizure, sleep, tremor, and urination and were monitored, and no adverse effects were observed in the test mice. Thus, the KP aerial part ethanol extract was relatively safe.

#### 3.5.2. The Organ-Body Index

Changes in the weight of internal organs are considered a sign of toxicity after exposure to toxic substances [59]. The heart, liver, spleen, kidneys, and lungs are the main organs affected by the metabolic reactions induced by toxic substances [60]. The organ-body index had almost no difference between the control group and the group of mice given the 5000 mg/kg ethanol extract in both male and female mice (*p* > 0.05) (Table 5). However, the spleen weight of male mice increased (*I* = 0.70 ± 0.17%) in comparison with control mice (*I* = 0.47 ± 0.05%) and the kidney weight of female mice increased (*I* = 1.24 ± 0.06%) as compared to the control group (*I* = 1.12 ± 0.05%) (*p* < 0.05).

#### 3.5.3. Hematological Parameters

Hematological indices were used to determine the negative effects of the extract on homeostasis-related functions in healthy mice. The results of this study showed that the mean of platelet volume (7.00 ± 0.40 fL) and platelet distribution width (8.13 ± 0.86%) was higher in male mice using the extract than that in control male mice. Similarly, in female mice with the extract, the MCHC index (27.17 ± 0.70 g/L) increased with higher amount than that of control mice (Table 6). It is revealed that the extract stimulated platelet formation in male mice, increased iron absorption, and increased hemoglobin in red blood cells in female mice. The remaining hematological parameters did not change, showing that the extract did not affect erythropoiesis as well as the immune system of test mice.

#### 3.5.4. Biochemical Parameters

Biochemical parameters suggested possible toxic effects of herbal medicines on liver and kidney function. Evaluation of liver and kidney function is very important because these two organs are mainly involved in body detoxification and homeostasis [61, 62].

When the membranes of the liver cells were damaged, many enzymes normally found in the cells are released into the bloodstream [63]. AST and ALT are liver enzymes that can often be used for screening and evaluating the liver function [64]. The results of this study showed no distinct changes in AST and ALT levels in test mice compared to control mice.

The quantification of renal function parameters in laboratory mice will directly determine the authorization of clinical trials of novel therapeutic agents as well as the licensing of a new medicine [65]. Serum uric acid levels were elevated with acute kidney injury and decreased glomerular filtration rate [66, 67]. Following the same pattern as the quantification of liver function, the results of the investigation of the toxic effects of the extract on the kidney through the uric acid index also did not show any difference between the toxic treatment and the control treatment.

Blood glucose values were used to assess the body's carbohydrate metabolism [68]. The concentration of toxic mice blood glucose levels was similar to that of control mice blood glucose (Table 7). From the above results, it is shown that the ethanol extract of KP at a dose of 5000 mg/kg did not affect liver and kidney function, as well as blood glucose in experimental mice.

#### 3.5.5. Histopathological Structure

Histological evaluation plays an important role in the assessment of organ damage associated with toxicity testing. It could provide information on the severity of injury, the prognosis of necrosis, fibrosis, and tissue inflammation to determine the degree of toxicity [68]. Therefore, the evaluation of histological features of different organs was performed in preclinical studies to determine drug side effects and toxicity.

**Figure 2:**
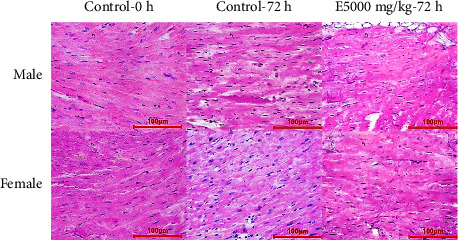
Histology of mice' heart tissue in treatments (H & E stain).

Examination of the myocardial tissue structure showed that cardiac muscle cells and connective tissues were in normal status. There was no sign of myocardial cell necrosis in the treatment using the ethanol extract with the dose of 5000 mg/kg in 72 h compared with the control treatment (Figure 2).

**Figure 3:**
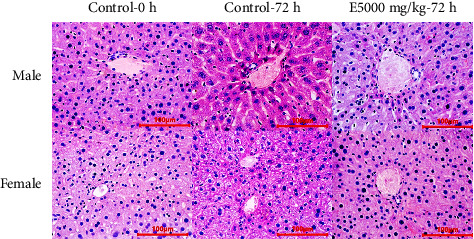
Liver tissue of mice in different treatments (H & E stain).

There were no histological changes in liver tissues of drug-tested mice. The liver cells are arranged in normal rows, no swelling, clearly visible vascular cysts. The liver tissue structure showed no signs of damage, necrosis, or hemorrhage (Figure 3).

**Figure 4:**
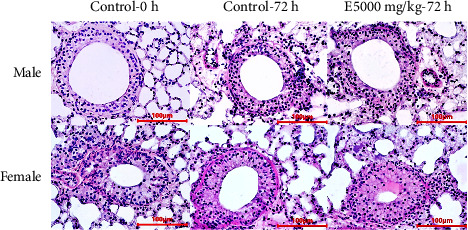
Histological structure of lung of mice in different treatments (H & E stain).

In lung tissues, no accumulation of alveolar edema, congestion, or inflammation of alveolar squamous cells was observed (Figure 4).

**Figure 5:**
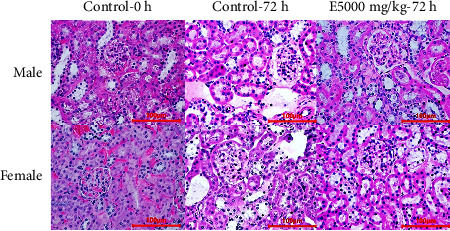
Histological structure of mice' renal tissues in different treatments (H & E stain).

Figure 5 shows that the renal tissue of mice drinking 5000 mg/kg extract remained normal, with no structural changes in the glomeruli, proximal tubule, and distal tubule in comparison with the control group drinking distilled water. There were no signs of necrosis or bleeding in the renal tissue.

**Figure 6:**
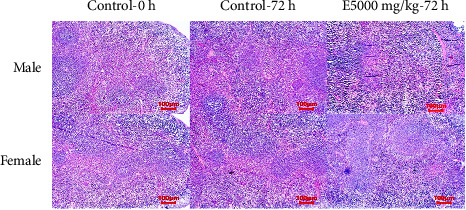
Histological structure of mice' spleen in different treatments (H & E stain).

The spleen is the organ that regulates the formation and destruction of red blood cells. The spleen has a role in regulating the number of red blood cells and other tangible elements of blood [69]. The results of histological examination demonstrated that the structure of the spleen tissue of the mice using the extract was similar to that of the control treatment (Figure 6).

**Figure 7:**
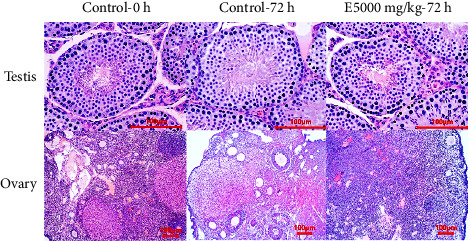
Histology of testis and ovary of mice in different treatments (H & E stain).

The effect of the extract on the reproductive system performance of mice was also evaluated through evaluation of gonadal tissue. Testicular tissues of control mice and mice taking the extract were clearly observed at different stages of spermatogenesis, Leydig cells developed normally, and no atrophy of the seminiferous tubules (Figure 7). The same was true for the ovaries, which showed that female mice given the extract had normal development of follicles compared to control mice (Figure 7).

The histological analysis of the organs was homologous and consistent with the biochemical indices analyzed above for liver and kidney function. This result adds to the basis of the safety profile of the extract at a dose of 5000 mg/kg body weight in mice.

## 4. Conclusion

Extracts from various sections of KL contained 19 physiologically active secondary metabolites. The ethanol extracts from the aerial parts of KL showed great antibacterial and antioxidant abilities. The extract was nontoxic at a dose of 5000 mg/kg body weight of mice and did not change the tissue structure and hematological and biochemical indices as compared to the control. KP is safe and has great potential in developing to make medicine to treat diseases (hepatitis, pharyngitis, and pneumonia) as mentioned in traditional remedies.

## Figures and Tables

**Table 1:** Chemical composition of KP.

Phytochemical	Extracts																			
	Methanol				Ethanol				Water				Diethyl ether				Chloroform			
	R	Rh	AP	W	R	Rh	AP	W	R	Rh	AP	W	R	Rh	AP	W	R	Rh	AP	W
Alkaloid	−	−	−	−	−	−	−	−	−	+	−	−	−	−	−	−	−	−	−	−
Carbohydrate	+	+	−	+	−	+	−	+	+	+	+	+	−	−	−	−	+	+	+	+
Cardiac glycoside	+	+	+	+	+	+	+	+	−	+	+	−	+	+	+	+	+	+	+	+
Flavonoid	+	+	+	+	−	+	+	+	+	+	+	+	+	+	−	−	−	−	+	−
Phenol	+	+	−	+	+	+	−	+	+	+	+	+	−	−	−	−	−	−	−	−
Protein/amino acid	−	−	−	+	−	−	+	+	−	−	+	−	−	−	+	−	−	+	−	−
Saponin	−	−	−	−	−	−	−	−	−	+	−	−	−	−	−	−	−	−	−	−
Sterol	+	−	−	−	−	−	−	−	−	−	−	−	−	−	−	−	−	+	−	−
Tannin	−	+	−	+	+	+	−	+	−	+	+	+	−	−	−	−	−	−	−	−
Terpenoid	−	−	−	−	−	−	−	−	−	−	−	−	−	−	−	−	−	+	+	−
Gum	−	−	−	−	−	−	−	−	−	−	−	−	−	−	−	−	+	+	−	−
Glycoside	+	−	−	−	−	+	−	−	−	−	−	−	−	−	−	−	+	−	−	−
Phlobatannin	−	−	−	−	−	−	−	−	−	−	−	−	−	−	−	−	−	−	−	−
Xanthoproteic	−	−	−	−	−	−	+	+	−	−	−	−	−	−	+	+	+	+	−	−
Anthocyanin	+	−	−	+	−	−	−	−	+	−	−	−	−	−	−	−	−	−	−	−
Coumarin	+	+	+	+	−	−	+	+	−	+	+	−	+	−	−	+	−	−	−	−
Essential oil	+	+	+	+	+	+	+	+	−	−	−	−	−	−	−	−	−	+	+	+
Diterpenes	+	−	+	+	+	−	+	+	+	−	+	+	−	−	−	−	−	−	−	−
Resins	−	−	+	+	−	−	+	+	−	−	−	−	+	−	+	+	−	−	−	−
Betalains	+	−	−	+	+	−	+	+	+	−	+	+	−	+	+	+	+	+	−	−

R: root; Rh: rhizome; AP: aerial parts; W: whole plants; (−): not present; (+): present.

**Table 2:** Extraction efficiency (%) from parts of KP.

Parts of the plant	Solvent		
	Methanol	Ethanol	Hot water
Root	2.27 ± 0.15^aB^	1.27 ± 0.06^aA^	2.70 ± 0.17^aC^
Rhizome	12.03 ± 0.23^dB^	4.27 ± 0.06^dA^	12.77 ± 0.51^bcB^
Aerial parts	10.90 ± 0.26^cB^	2.60 ± 0.17^bA^	15.23 ± 0.93^cC^
Whole plant	10.27 ± 0.15^bB^	2.87 ± 0.06^cA^	10.73 ± 0.95^bB^

Uppercase letters in a row and lowercase letters in the same column are not statistically significant (Duncan, *p* ≥ 0.05).

**Table 3:** EC_50_ value of antioxidant activities from different parts of KP.

No.	Extract	*n*	EC_50_ (*μ*g/mL)	Regression equation
1	VitaC	9	2.00 ± 0.04^a^	*y* = 26.718*x* − 3.4527 (*R*^2^ = 0.9897)
2	REt	9	15.03 ± 0.43^c^	*y* = 3.2886*x* + 0.5976 (*R*^2^ = 0.9793)
3	RMe	9	9.54 ± 0.37^b^	*y* = 4.3974*x* + 8.0772 (*R*^2^ = 0.9743)
4	RHw	9	118.20 ± 2.66^m^	*y* = 0.3975*x* + 3.0454 (*R*^2^ = 0.9848)
5	RhEt	9	20.86 ± 0.48^d^	*y* = 2.5873*x* − 3.946 (*R*^2^ = 0.9718)
6	RhMe	9	80.11 ± 1.29^i^	*y* = 0.5586*x* + 5.2597 (*R*^2^ = 0.9793)
7	RhHw	9	103.50 ± 1.25^k^	*y* = 0.428*x* + 5.702 (*R*^2^ = 0.979)
8	APEt	9	34.07 ± 1.06^g^	*y* = 1.408*x* + 2.0793 (*R*^2^ = 0.9846)
9	APMe	9	107.97 ± 2.95^l^	y = 0.424*x* + 4.2529 (*R*^2^ = 0.9803)
10	APHw	9	145.21 ± 3.11^n^	*y* = 0.3419*x* + 0.3765 (*R*^2^ = 0.9826)
11	WEt	9	27.38 ± 0.62^f^	*y* = 1.6625*x* + 4.4992 (*R*^2^ = 0.9706)
12	WMe	9	76.24 ± 3.60^h^	*y* = 0.5856*x* + 5.4139 (*R*^2^ = 0.9786)
13	WHw	9	94.75 ± 2.92^j^	*y* = 0.4809*x* + 4.4609 (*R*^2^ = 0.9794)

VitaC: vitamin C; REt: root ethanol, RMe: root methanol; RHw: root hot water; RhEt: rhizome ethanol; RhMe: rhizome methanol; RhHw: rhizome hot water; APEt: aerial parts ethanol; APMe: I aerial parts methanol; APHw: aerial parts hot water; WEt: whole plant ethanol; WMe: whole plant methanol; WHw: whole plant hot water. Means ± SD having the same letter in a column are not significantly different (Duncan, *p* ≥ 0.05).

**Table 4:** Antibacterial diameter of KP extracts at a concentration of 200 mg/mL.

Extracts	Antibacterial ring diameter (mm)							
	Gram-positive				Gram-negative			
	*S. aureus*	*L. innocua*	*B. subtilis*	*B. cereus*	*E. faecalis*	*P. aeruginosa*	*Salmonella*	*E. coli*
REt	14.17 ± 0.29^fC^	11.00 ± 1.00^gAB^	11.83 ± 1.04^gB^	11.83 ± 0.29^hB^	13.17 ± 0.29^efC^	13.33 ± 0.58^gC^	13.67 ± 0.58^fgC^	10.33 ± 0.58^fA^
RMe	14.00 ± 0.00^fE^	11.17 ± 0.29^gA^	11.67 ± 0.58^gAB^	12.33 ± 0.58^hBC^	13.00 ± 0.50^eCD^	13.33 ± 0.58^gDE^	14.00 ± 0.00^gE^	11.17 ± 0.29^fgA^
RHw	0.00 ± 0.00^aA^	0.00 ± 0.00^aA^	4.67 ± 0.58^cdBC^	5.50 ± 0.87^eC^	3.83 ± 0.29^bB^	14.17 ± 0.76^fgE^	9.17 ± 0.76^cdeD^	4.50 ± 0.00^cB^
RhEt	6.83 ± 0.29^dA^	9.67 ± 0.58^fBC^	9.67 ± 0.58^fBC^	8.67 ± 1.15^gB^	9.67 ± 0.58^dBC^	9.33 ± 0.58^fBC^	8.33 ± 1.53^cdeB^	10.67 ± 0.58^fC^
RhMe	0.00 ± 0.00^aA^	0.00 ± 0.00^aA^	4.00 ± 0.50^bcB^	4.17 ± 0.29^cdB^	4.33 ± 0.29^bB^	6.17 ± 0.29^deC^	6.67 ± 0.29^bcC^	4.50 ± 0.50^cB^
RhHw	4.00 ± 0.00^cAB^	5.67 ± 0.58^dCDE^	3.83 ± 0.29^bcA^	6.00 ± 0.00^efDE^	6.33 ± 0.58^cE^	5.33 ± 0.58^cdCD^	4.83 ± 0.76^bBC^	5.33 ± 0.58^cdCD^
APEt	6.83 ± 0.28^dA^	10.67 ± 1.53^fgB^	6.17 ± 0.29^deA^	6.67 ± 1.15^fA^	6.17 ± 0.76^cA^	7.33 ± 1.54^eA^	10.00 ± 0.00^deB^	6.33 ± 0.58^deA^
APMe	2.33 ± 0.58^bcA^	2.17 ± 0.29^bA^	3.17 ± 0.29^bB^	4.17 ± 0.29^cdC^	4.67 ± 0.58^bCD^	5.00 ± 0.00^cdD^	9.67 ± 0.76^cdeE^	4.33 ± 0.29^bcCD^
APHw	3.50 ± 0.50^cB^	0.00 ± 0.00^aA^	5.00 ± 1.00^cdD^	3.67 ± 0.29^bcBC^	4.50 ± 0.00^bCD^	3.33 ± 0.58^bB^	7.67 ± 0.58^bcdE^	3.33 ± 0.58^bB^
WEt	0.00 ± 0.00^aA^	7.67 ± 0.58^eD^	7.17 ± 0.29^eCD^	8.50 ± 0.50^gE^	6.83 ± 0.29^cC^	7.17 ± 0.29^eCD^	1.33 ± 0.58^aB^	6.83 ± 0.29^eC^
WMe	0.00 ± 0.00^aA^	0.00 ± 0.00^aA^	5.00 ± 0.00^cdC^	5.00 ± 0.00^deC^	4.17 ± 0.29^bB^	5.17 ± 0.29^cdC^	7.00 ± 1.00^bcdD^	4.83 ± 0.29^cBC^
WHw	0.00 ± 0.00^aA^	0.00 ± 0.00^aA^	5.00 ± 0.00^cdC^	0.00 ± 0.00^aA^	0.00 ± 0.00^aA^	0.00 ± 0.00^aA^	7.67 ± 0.58^bcdD^	3.33 ± 0.29^bB^
Amox	1.67 ± 1.15^abAB^	3.17 ± 0.76^bcBC^	0.00 ± 0.00^aA^	2.83 ± 0.29^bBC^	4.17 ± 0.76^bC^	4.00 ± 1.00^bcC^	37.00 ± 1.73^kD^	0.67 ± 0.58^aA^
Tetra	10.33 ± 1.54^eAB^	11.33 ± 1.53^gB^	13.33 ± 0.76h^B^	12.83 ± 0.29^hB^	14.00 ± 0.87^fB^	14.00 ± 1.80^fgB^	18.00 ± 5.00^hC^	7.33 ± 0.58^eA^
Eryth	10.00 ± 1.00^eA^	16.00 ± 1.00^hB^	34.00 ± 1.00^jD^	35.17 ± 1.04^kD^	34.83 ± 0.76^iD^	15.17 ± 0.76^gB^	25.33 ± 2.31^jC^	27.33 ± 1.15^iC^
Linco	29.00 ± 2.65^gF^	3.67 ± 0.58^cA^	21.67 ± 2.31i^C^	26.33 ± 0.58^jE^	25.17 ± 1.04^hDE^	22.83 ± 0.29^hCD^	11.17 ± 2.02^efB^	24.50 ± 0.5^hDE^
Strep	27.67 ± 2.08^gD^	11.33 ± 0.58^gA^	21.67 ± 1.04i^B^	24.17 0.76^iC^	22.00 ± 0.05^gB^	23.00 ± 1.00^hBC^	21.33 ± 0.58^iB^	12.00 ± 1.32^gA^

REt: root ethanol; RMe: root methanol; RHw: root hot water; RhEt: rhizome ethanol; RhMe: rhizome methanol; RhHw: rhizome hot water; APEt: aerial part ethanol; APMe: aerial part methanol; APHw: aerial parts hot water; WEt: whole plant ethanol; WMe: whole plant methanol; WHw: whole plant hot water; Amox: amoxicillin (1 mg/mL); tetra: tetracycline (1 mg/mL); Eryth: erythromycin (1 mg/mL); Linco: lincomycin (1 mg/mL); Strep: streptomycin (1 mg/mL); mean diameter ± SD with the same uppercase letters in 1 row and the same lowercase letters in 1 column was not statistically significant (*p* > 0.05).

**Table 5:** Effects of KP extracts to organ-body indices.

Sex	Organ	The organ-body index (%)		
		Control-0 h	Control-72 h	E5000 mg/kg-72 h
Male	Heart	0.47 ± 0.08^a^	0.49 ± 0.04^a^	0.47 ± 0.09^a^
	Liver	4.27 ± 0.32^a^	4.28 ± 0.22^a^	4.83 ± 0.39^a^
	Lungs	0.76 ± 0.02^a^	0.76 ± 0.03^a^	1.09 ± 0.19^b^
	Kidney	1.29 ± 0.15^a^	1.45 ± 0.23^a^	1.30 ± 0.11^a^
	Spleen	0.47 ± 0.05^a^	0.47 ± 0.07^a^	0.70 ± 0.17^b^
	Testis	0.62 ± 0.21^a^	0.71 ± 0.23^a^	0.69 ± 0.08^a^

Female	Heart	0.44 ± 0.08^a^	0.53 ± 0.02^a^	0.44 ± 0.04^a^
	Liver	4.57 ± 0.40^a^	4.93 ± 0.14^a^	4.75 ± 0.59^a^
	Lungs	1.01 ± 0.17^a^	0.98 ± 0.14^a^	1.24 ± 0.25^a^
	Kidney	1.12 ± 0.05^a^	1.15 ± 0.07^ab^	1.24 ± 0.06^b^
	Spleen	0.62 ± 0.15^a^	0.51 ± 0.08^a^	0.46 ± 0.05^a^
	Ovary	0.05 ± 0.02^a^	0.06 ± 0.01^a^	0.06 ± 0.02^a^

Means ± SD with different letters in a row are statistically significant (Duncan, *p* < 0.05).

**Table 6:** Effect of KP extract on hematological indices.

Sex	Indices	Control-0 h	Control-72 h	E5000 mg/kg-72 h
Male	RBC (10^12^/L)	8.61 ± 0.52	8.78 ± 0.80	8.23 ± 0.30
	HGB (g/dL)	13.23 ± 0.93	12.87 ± 0.49	12.43 ± 0.40
	HCT (L/L)	49.43 ± 4.48	49.80 ± 3.22	46.97 ± 1.29
	MCV (fL)	57.40 ± 2.10	56.90 ± 4.07	57.07 ± 1.63
	MCH (pg)	15.40 ± 0.61	14.73 ± 1.37	15.13 ± 0.32
	MCHC (g/L)	26.87 ± 0.90	25.87 ± 0.93	26.47 ± 0.29
	PLT (G/L)	1115.33 ± 179.45	828.00 ± 203.65	823.00 ± 115.88
	PCT (L/L)	0.73 ± 0.12	0.54 ± 0.13	0.58 ± 0.10
	MPV (fL)	6.53 ± 0.06	6.47 ± 0.33	7.00 ± 0.40^*∗*^
	PDW (%)	7.33 ± 0.35	6.57 ± 0.25	8.13 ± 0.86^*∗*^
	WBC (10^9^/L)	8.10 ± 1.92	8.01 ± 3.27	5.70 ± 0.70
	NEU (%)	0.39 ± 0.19	0.23 ± 0.14	0.22 ± 0.08
	LYM (%)	7.43 ± 1.62	7.45 ± 3.09	5.29 ± 0.63
	MON (%)	0.08 ± 0.05	0.15 ± 0.06	0.10 ± 0.06
	EOS (%)	0.00 ± 0.00	0.00 ± 0.00	0.00 ± 0.00
	BAS (%)	0.19 ± 0.08	0.16 ± 0.09	0.08 ± 0.06

Female	RBC (10^12^/L)	8.27 ± 0.23	9.04 ± 0.51	8.77 ± 0.62
	HGB (g/dL)	13.00 ± 0.17	13.37 ± 0.81	13.47 ± 0.85
	HCT (L/L)	47.37 ± 0.93	51.27 ± 2.80	49.53 ± 1.91
	MCV (fL)	57.27 ± 0.51	56.70 ± 0.89	56.60 ± 2.88
	MCH (pg)	15.70 ± 0.36	14.80 ± 0.30	15.37 ± 0.74
	MCHC (g/L)	27.43 ± 0.40	26.13 ± 0.25^*∗*^	27.17 ± 0.70
	PLT (G/L)	721.33 ± 13.58	773.33 ± 102.34	668.67 ± 124.46
	PCT (L/L)	0.48 ± 0.04	0.50 ± 0.06	0.45 ± 0.06
	MPV (fL)	6.67 ± 0.46	6.47 ± 0.21	6.87 ± 0.64
	PDW (%)	7.30 ± 1.06	6.77 ± 0.57	7.53 ± 1.55
	WBC (10^9^/L)	6.11 ± 2.15	6.67 ± 0.63	4.52 ± 2.10
	NEU (%)	0.19 ± 0.07	0.17 ± 0.06	0.24 ± 0.07
	LYM (%)	5.69 ± 2.08	6.35 ± 0.59	4.08 ± 1.96
	MON (%)	0.07 ± 0.04	0.03 ± 0.02	0.07 ± 0.01
	EOS (%)	0.01 ± 0.01	0.00 ± 0.00	0.00 ± 0.00
	BAS (%)	0.13 ± 0.06	0.12 ± 0.05	0.11 ± 0.08

RBC: red blood cell; HGB: hemoglobin; HCT: hematocrit; MCV: mean cell volume; MCH: mean cell hemoglobin; MCHC: mean cell hemoglobin concentration; PLT: platelet count; PCT: procalcitonin; MPV: mean platelet volume; PDW: platelet distribution width; WBC: white blood cell; NEU: neutrophils; LYM: lymphocytes; MON: monocytes; EOS: eosinophils; BAS: basophils. Mean ± SD having ^*∗*^ is significantly different from others in the same row (Duncan, *p* < 0.05).

**Table 7:** Effect of KP extract on biochemical indices.

Sex	Biochemical	Control-0 h	Control-72 h	E5000 mg/kg-72 h
Male	Glucose blood (mg/dL)	161.33 ± 35.13	150.33 ± 26.63	172.33 ± 32.58
	AST (U/L)	59.33 ± 12.70	45.00 ± 7.21	45.33 ± 6.11
	ALT (U/L)	30.67 ± 9.61	21.67 ± 1.53	24.33 ± 8.50
	Uric acid (*μ*mol/L)	175.00 ± 26.89	141.00 ± 28.05	146.33 ± 46.46

Female	Glucose blood (mg/dL)	158.33 ± 24.09	159.33 ± 12.50	161.33 ± 32.33
	AST (U/L)	82.67 ± 19.55	65.67 ± 19.50	60.67 ± 9.45
	ALT (U/L)	20.00 ± 3.61	30.33 ± 15.95	20.67 ± 5.51
	Uric acid (*μ*mol/L)	138.67 ± 28.31	123.00 ± 20.66	158.33 ± 49.64

Data: means ± SD. There is not enough evidence that there is a difference among treatments (Duncan, *p* ≥ 0.05).

## Data Availability

Raw and analyzed data of the study will be made available upon request.
